# Facilitators of tertiary students' entrepreneurial intentions: Insights for Lesotho's national entrepreneurship policy

**DOI:** 10.1016/j.heliyon.2023.e17511

**Published:** 2023-06-22

**Authors:** Christopher Dick-Sagoe, Ka Yiu Lee, Augustine Osei Boakye, Kofi Nkonkonya Mpuangnan, Peter Asare-Nuamah, Anna Dankwaah Dick-Sagoe

**Affiliations:** a Political and Administrative Studies Department, University of Botswana, Gaborone, Botswana; b Department of Health Sciences, Mid Sweden University, Ostersund, Sweden; c Department of Management and Human Resource, Ghana Communication Technology University, Accra, Ghana; d Department of Curriculum and Instructional Studies, University of Zululand, South Africa; e Center for Development Research, University of Bonn, Bonn, Germany; f School of Sustainable Development, University of Environment and Sustainable Development, Somanya, Eastern Region, Ghana; g Enko Botho, Gaborone, Botswana

**Keywords:** Entrepreneurial intentions, Entrepreneurship education, Facilitators of entrepreneurship, National university of Lesotho, Theory of planned behaviour

## Abstract

This study provides insightful information to guide the Lesotho government's drive to address rising youth unemployment. Through quota sampling technique, this study selected 930 students from 31 departments at National University of Lesotho. Grounded in the theory of planned behaviour (TPB), the study examined the facilitators of students' entrepreneurial intentions using mean, standard deviation, mean rank, correlation, and Mann-Whitney *U* test. Structural equation modelling was used to determine the relationship between the three components of TPB (attitudes, perceived behavioural control, and subjective norm), and students' entrepreneurial intention. The findings show that attitudes and perceived behavioural control were positive predictors of entrepreneurial intention while subjective norm was a negative predictor. The major findings indicate that students from Business and Management Development, Business Administration, Economics, Nutrition, and Pharmacy departments had higher entrepreneurial intentions, with postgraduate students (at master level) having higher entrepreneurial decisions than undergraduate students. Policy, practice, and research implications are teased out from the findings to improve entrepreneurial education.

## 1 Introduction

University education plays a crucial role in the socio-economic development of every nation. One of the most important goals of university education is to empower students with employable skills necessary to improve their living conditions and contribute to national development [1]. Therefore, entrepreneurial education in universities can serve as a conduit to increase entrepreneurial intentions among graduate students by acquiring the necessary skills, abilities, and attitudes needed in entrepreneurship [2].

The overall high literacy rate in Lesotho of around 90% probably means that successive governments have made efforts to provide education to their people [3,4]. Nonetheless, the country faces dwindling local market due to entrepreneurs and skilled labour scarcity [5]. Lesotho currently has high youth unemployment rate of about 45 to 50% [6], resulting in poverty and economic stagnation [7,8]. Youth unemployment is particularly high among university graduates [4,9], making entrepreneurship an alternative solution to the troubling situation. According to the United Nations Development Programme and the International Development Research Centre on Entrepreneurship, the Lesotho government has resorted to a youth entrepreneurship programme as a panacea to youth unemployment, crime, poverty, and economic stagnation [4]. Indeed, the available statistics indicates that only 7% of Lesotho's youth have started their own businesses [4]. However, while 49% of Lesotho's youth have entrepreneurial interest, factors such as low skills, capital, and access to information hinder youth entrepreneurship [4,9].

**Fig. 1:**
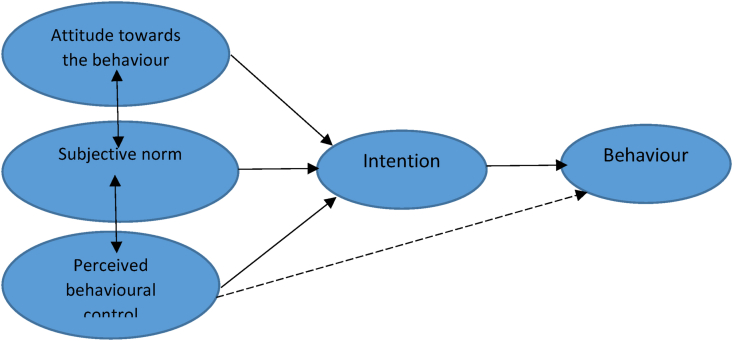
The theory of planned behaviour.

Source: Ajzen & Madden (1986)

Although several authors have studied entrepreneurship and generated models that predict entrepreneurial intentions [10,11], yet, entrepreneurship education has not gone well with the citizens of Lesotho. Graduates are not demonstrating entrepreneurial intentions and competencies in Lesotho [12]. The reason is that Lesotho's education adopts a neoliberal human capital approach and does not create better skilled workers [13]. This is in stark contrast to the idea that education should prepare youth to be entrepreneurial, problem solvers, and capable of taking independent decisions and initiatives [14]. Therefore, a question arises as to what drives students' entrepreneurial intentions at the National University of Lesotho? The answer to such a question is crucial for enhancing economic planning and preparing a skilled workforce for national development. The National Strategic Plan II (2018/19–2022/23) of the Kingdom of Lesotho states that youth unemployment, together with its social-economic consequences, remains a huge national development challenge fuelled by inadequate technical skills, limited work experience, and a lack of entrepreneurial skills coupled with the absence of avenues to provide skills, ideas, and information. Given the absence of an avenue for sharing entrepreneurial skills and knowledge, which is critical for stimulating entrepreneurial intentions among Lesotho youth, this study, grounded in the Theory of Planned Behaviour (TPB) (Fig. 1), is carried out as an attempt to broaden an understanding on how state institutions can exploit avenues for entrepreneurial education.

The TPB has been widely used in behavioural studies in many fields [15]. Specific to entrepreneurial intention, the literature highlights that attitude exerts a greater influence on entrepreneurial intention than perceived behaviour control [16]. As a result [17], hypothesized that a third variable could moderate the relationship between attitudes, behaviour, and intention. Also, Conner [18] opined that perceived behaviour control can influence the attitudes of people who portray positive attitudes and supportive subjective norms toward the behaviour. In addition, Williams, He and Conners [19] argued that the higher the level of positive attitude people demonstrate, the higher their subjective norms. The authors further argue that performance is largely determined by a plan of behaviour. This can be determined by a situation where a person portrays an attitude of readiness to perform or not perform an innate behaviour. Although the TPB is well-known for improving decision-making, it can also be self-motivated and regulated by the person(s) in question [20].

This study is relevant due to high graduate unemployment amidst successive governments' efforts to address youth unemployment through the National Youth Policy, which promotes entrepreneurial intentions. It is problematic to note that studies on entrepreneurial intentions among university students, particularly in Lesotho's only public university, are either scanty or lacking. This study serves to fill the identified gap, contribute to government efforts towards promoting entrepreneurial intention among students, and guide further studies. Based on the extant literature, the present study answers the following research questions:

1) What is the relationship between university programmes and students' entrepreneurship decision?
2) Do students' educational level influence their entrepreneurial decisions?

These research questions serve to guide the achievement of the goal of the study, which seeks to ascertain the facilitators of tertiary students' entrepreneurial intentions from the National University of Lesotho.

### 1.1 Theoretical foundation: theory of planned behaviour

The TPB is derived from the theory of reasoned action (TRA) [21]. TPB argues that certain factors predict a person's behaviour and attitude. One such factor is a person's intentions. Williams, He and Conners [19] assert that the more people demonstrate a positive attitude, the stronger their subjective norms, and the more perceive control over their behaviour, the more likely they will perform the proposed behaviour. For this reason, it can be said that the TPB operates on three factors: attitudes, subjective norms, and perceived behavioural control, as shown in Fig. 1.

Three hypotheses are further postulated to steer the affair of the study:

• Hypothesis 1: Attitude has a significant positive relationship with students' entrepreneurial intentions.
• Hypothesis 2: Subjective norms has a significant negative relationship with students' entrepreneurial intentions.
• Hypothesis 3: Perceived behavioural control has a significant positive relationship with students' entrepreneurial intentions.

We argue that if the students at the National University of Lesotho (NUL) believe that entrepreneurship is a good idea, there is a higher likelihood that they will make effort to acquire the needed knowledge, skills, and experience. Subjective norms refer to the views and beliefs of society pertaining to a particular behaviour [22]. Social interaction and the belief system in a society have the potential to shape the behaviour of the members of that society [23].

Indeed, subjective norms have a tremendous influence on students who intend to venture into entrepreneurship. The questions that frequently baffle the minds of such students are: Will society approve or disapprove a positive attitude towards entrepreneurial activity? What will society think if a student starts an entrepreneurial activity? Will graduates in entrepreneurship be considered taboo? How will the reputation of the graduate student be affected if society finds out that s/he has the intention of starting or has started an entrepreneurial activity? These questions, therefore, determine the strength of the students’ entrepreneurial intention.

Perceived behavioural control connotes the ability to execute an intended behaviour [24] and is determined by examining the present condition of people to know whether they have the skillsets and equipment to perform a given behaviour. Perceived behavioural control suggests that venturing into entrepreneurship can be an easy or difficult task for students, depending on their level of training. In assessing the perceived behavioural control of students, the questions that pop up include: how long does it take for students to develop their entrepreneurial intention? Do students have the necessary amount of capital to start a venture? As posited by Lee and Wong [25], the attitude of students towards entrepreneurship is developed in their early ages, at about twelve years of age. At this age, students are explorative while studying at basic school, where they have a high inclination to learn new things. This means introducing students to skills at an early stage would expose them to entrepreneurship.

Previous research has established evidence on models of entrepreneurship education at the university level [26]. Entrepreneurial teaching models, which tend to provide students with a degree of control over the entrepreneurial environment, also strongly impacts students' entrepreneurial intentions. For instance, models of training that involve the invitation of renowned entrepreneurs to interact with students tend to give students the urge to walk over subjective societal norms that oppose entrepreneurial intentions. In addition, evidence suggests that students’ intention to start entrepreneurship as an income-generating activity is based on factors such as attitudes, perceived control, and subjective norms [22]. The present study, therefore, aims at examining the facilitators of students' entrepreneurial intentions at the NUL in the context of the TPB model.

## 2 Methodology

The National University of Lesotho (NUL) approved this study, and informed consent was obtained from all participants. This study adopted a descriptive survey design and inductive reasoning approaches. This design involved the measurement of a particular population's characteristics over a certain period [27], which is useful for measuring students' entrepreneurial intentions in entrepreneurship training schedules. The study sample comprised final-year undergraduate and master's degree students of the NUL. A quota sampling technique was used to select a total of 930 students, comprising 620 undergraduates (20 from each department) and 310 master students (10 from each department).

The study used a self-administered questionnaire, which was divided into five sections: biodata (Section A), attitude towards entrepreneurship (Section B), subjective norm towards entrepreneurship (Section C), perceived behavioural control over the ability to study entrepreneurship (Section D), and entrepreneurial intention (Section E). The questions were adapted from Silva et al. [11] and the framework developed by the Global University Entrepreneurial Spirit Students’ Survey (GUESSS). With the exception of section, A, a 5-point Likert scale (Strongly Agree-5, Agree-4, Neutral-3, Disagree-2, and Strongly Disagree-1) was used. In the analysis, mean and standard deviation (SD) were used with values above 3.0 (5 + 4+3 + 2+1/5 = 3.0) showing majority of the respondents in favour of the statement whiles a mean value below 3.0 showed majority of the respondents were not in favour of the statement. Moreover, the overall mean and p-value (significant at 0.05) were used in comparing mean and making additional judgment about the collected data, which is consistent with a previous study by Munyanyiwa et al. [28].

Two pilot tests were conducted to ensure that it accurately measured the intended constructs and enhanced the instrument's content and face validity [29]. The self-administered questionnaire was sent to 1000 students from September to October 2020 through Google Forms. A total of 930 students returned the questionnaire. The study also complied with voluntary participation and anonymity, and the participants had the right to terminate the study at any time.

**Fig. 2:**
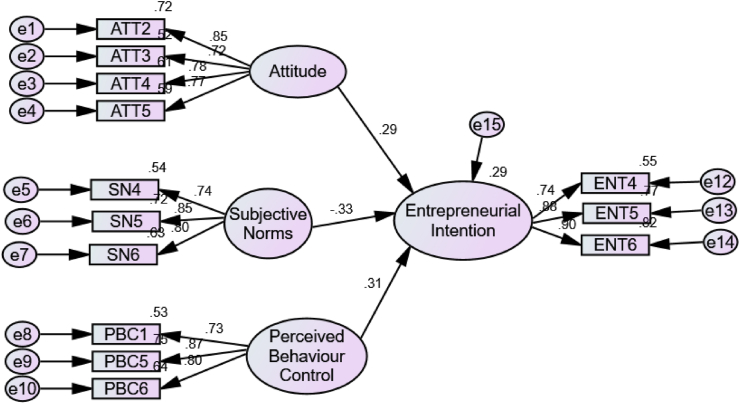
**Structural model predicting entrepreneural intentions.** Estimated using AMOS 21.

Preliminary analyses were conducted to identify outliers, and test data normality. In addition, the reliability of the data was examined through Cronbach's alpha computation [37]. The reliability coefficients for different categories were 0.83 (attitude), 0.84 (subjective norm), 0.84 (perceived behaviour control) and 0.88 (entrepreneurial intention), which indicated high internal reliability of the instrument. Both basic descriptive and inferential statistical analyses were performed. The authors used the Mann-Whitney *U* test to examine the difference between the medians of two independent groups. Spearman's rank correlation coefficient was also calculated to determine the relationships between the independent variables (attitude, subjective norms, and perceived behavioural control) and the dependent variable (entrepreneurial intentions). In addition, a structural equation model (SEM) was computed to understand the drivers of students' entrepreneurial intentions. The analyses were conducted using SPSS version 21 and AMOS version 21. Structural equation modelling (SEM) was used to determine the relationship among the three components of TPB, namely, attitudes, subjective norms, perceived behavioural control, and intention towards entrepreneurship as can be seen in Fig. 2. AMOS version 21 was used as SEM analytical tool to test the strength and the direction of the purported relationships. Ethical clearance for the study was obtained from the Ethics Committee of the National University of Lesotho's Institutional Review Board (NUL/EXT/2022/01).

## 3 Findings and discussion

The results are presented in two sections. The first section focuses on students' entrepreneurship decisions based on university programmes. The second section focuses on the influence of students’ educational level on their entrepreneurial decisions, and the contributions of subjective norms, perceived behavioural control, and attitude to entrepreneurial intentions.

### 3.1 Section 1: Students’ decision on entrepreneurship based on NUL programmes

This section presents students' decisions on entrepreneurship based on various faculties and departments in the NUL. Table 1 shows the frequency and percentage of students' entrepreneurial intention among seven faculties and their respective departments. According to Table 1, students pursuing Development Studies (56.6%, mean = 4.87, SD = 0.72), Theology and Religious Studies (50%, mean = 4.50, SD = 0.65) and African Languages and Literature (36.6%, mean = 3.33, SD = 0.49) have a high entrepreneurial intention. Inversely, students in English (30%, mean = 2.71, SD = 0.39) and French (23.3%, mean = 2.14, SD = 0.29) departments had low entrepreneurial intentions while those in Historical Studies (10%, mean = 0.95, SD = 0.032) and Philosophy (16.6%, mean = 1.55, SD = 0.16) had the lowest entrepreneurial intentions. This implies that the curriculum of most of the departments in the Humanities does not teach skills needed to boost entrepreneurial interest among students. Therefore, such students may lack ideas and may not necessarily engage in entrepreneurship. However, Edelstein [30] found out that ‘‘the humanities play a determining role in producing not only the “right” kind of doctor, but also the entrepreneurs, engineers, and designers that make the American economy so productive’‘. Arguably, the difference may be attributed to the differences in the economies in the countries of choice for the two studies.

**Table 1:** Frequency and percentage of students’ Entrepreneurial intention.

Faculty	Department	N	%	Mean	Std. Deviation
Humanities	Development Studies	30	56.6	4.87	0.72
	Theology and Religious Studies	30	50.0	4.50	0.65
	African Languages and Literature	30	36.6	3.33	0.49
	English	30	30.0	2.71	0.39
	French	30	23.3	2.14	0.29
	Philosophy	30	16.6	1.55	0.16
	Historical Studies	30	10.0	0.95	0.03
Education	Educational Foundations	30	40.0	3.63	0.54
	Science Education	30	33.3	3.04	0.45
	Languages and Social Education	30	30.0	2.70	0.39
Health Sciences	Nutrition	30	70.0	4.98	0.85
	Pharmacy	30	63.3	4.90	0.79
	Environmental Health	30	53.3	4.84	0.69
	Nursing	30	43.3	3.97	0.58
Law	Private Law	30	26.6	2.47	0.35
	Public law	30	23.3	2.18	0.30
	Procedural and Adjectival Law	30	16.6	1.55	0.16
Science & Technology	Physics and Electronics	30	60.0	4.90	0.76
	Chemistry and Chemical Technology	30	56.6	4.70	0.72
	Mathematics and Computer Science	30	50.0	4.59	0.66
	Geography and Environmental Science	30	40.0	3.62	0.54
	Biology	30	30.0	2.79	0.41
	Business Administration	30	80.0	5.00	0.94
Social Sciences	Economics	30	70.0	4.99	0.85
	Political & Administrative Studies	30	60.0	4.50	0.76
	Sociology, Anthropology & Social Work	30	46.6	4.29	0.63
	Statistics & Demography	30	36.6	3.38	0.50
Institute of Extra Mural Studies (IEMS)	Business and Management Development	30	90.0	5.00	0.92
	Research, Evaluation and Media	30	60.0	4.49	0.76
	Non-Formal and Continuing Education	30	43.3	3.91	0.58
	Adult Education	30	23.3	2.19	0.30

Concerning the faculty of Education, students in the department of Educational Foundations (40%, mean = 3.63, SD = 0.54) expressed higher entrepreneurial decisions, whereas students in the department of Science Education (33.3%, mean = 3.04, SD = 0.45), and Languages and Social Education (30%, mean = 2.70, SD = 0.39) expressed otherwise. It is obvious that various departments of education run courses that are appropriate for equipping students with subject knowledge and pedagogy to be effective in the classroom. This means that such departments may not have incorporated elective courses that would motivate students to grasp entrepreneurial ideas, which is consistent with Hahn, Minola and Bosio [31].

In the faculty of Health Sciences, students in departments such as Nutrition (70%, mean = 4.98, SD = 0.85), Pharmacy (63.3%, mean = 4.90, SD = 0.79), and Environmental Health (53.3%, mean = 4.84, SD = 0.69) have high entrepreneurial decisions, while Nursing students (43.3%, mean = 3.97, SD = 0.58) have lower entrepreneurial decisions. The findings suggest that students in specific departments are motivated by some of their courses relating to entrepreneurship. It could be possible that the competencies required for being an effective health professional are synonymous with entrepreneurial competencies, as argued by Hatef, Sharfstein and Labrique [32].

Moreover, within the faculty of Law, students in the departments of Private Law (26.6%, mean = 2.47, SD = 0.35), Procedural and Adjectival Law (16.6%, mean = 2.18, SD = 0.30), and Public Law (23.3%, mean = 1.55, SD = 0.16) showed low entrepreneurial decisions. According to Ghadas, Herna and Hamid [33] prospective lawyers require entrepreneurial skills to set up businesses and firms like construction and trading companies. Such law firms combine modern technology to save time, money, and reduce risk. Despite the need for entrepreneurial skills, it can be learnt from the data that most of the courses that prepare students at the faculty of Law do not stimulate entrepreneurial intention among them.

Within the faculty of Science and Technology, students in the various departments have different entrepreneurial decisions. The department with the highest percentage of entrepreneurial decisions was Physics and Electronics (60.0%, mean = 4.90, SD = 0.76), followed by Chemistry and Chemical Technology (56.6%, mean = 4.70, SD = 0.72) and Mathematics and Computer Science (50.0%, mean = 4.59, SD = 0.66). However, students in the department of Geography and Environmental Science (40.0%, mean = 3.62, SD = 0.54) and Biology (30.0%, mean = 2.79, SD = 0.41) have low entrepreneurial decisions. It is believed that Science, Technology, and Entrepreneurship complement each other [34]. To sustain this relationship, experts from the field of entrepreneurship are invited occasionally to the Science faculty to revitalize business ideas and skills among the students [35]. Since the data from most of the students studying in various departments of Science and Technology show a high level of entrepreneurial decision-making, it suggests that most of them would know how to improve their lives by using scientific knowledge after completing university.

In the faculty of Social Sciences, most of the students have high levels of entrepreneurship decisions. The department with the highest percentage of entrepreneurs was Business Administration (80.0%, mean = 5.00, SD 0.94), followed by Economics (70.0%, mean = 4.99, SD 0.85), Political and Administrative Studies (60.0%, 4.50, SD = 0.76), Sociology, Anthropology, and Social Work (46.6%, mean = 4.29, SD = 0.63), and Statistics and Demography (36.6%, mean = 3.38, SD = 0.50). These findings corroborate that of Ramos [36] which showed that business students have a higher intention of venturing into entrepreneurship due to knowledge, ideas, and skills acquired from their line of studies. The higher percentage of students’ entrepreneurial decisions at various departments affirms the assertions made by previous studies. Therefore, the findings support that the business departments have incorporated courses that are appropriate for equipping students with entrepreneurial ideas.

Concerning the Institute of Extra Mural Studies (IEMS), it was found that students in the department of Business and Management Development (90.0%, mean = 5.00, SD = 0.92), Research, Evaluation, and Media (60.0%, mean = 4.49, SD = 0.76), and Non-Formal and Continuing Education (43.3%, mean = 3.90, SD = 0.58), have high entrepreneurial decisions, while those in Adult Education (23.3%, mean = 2.19, SD = 0.30) showed otherwise. Though there is a need to examine the courses in the department of Adult Education, the findings show that the IEMS has appropriate programmes that empower students to consider entrepreneurship.

The present study shows that students in departments like Business and Management Development, Business Administration, Economics, Nutrition and Pharmacy have higher entrepreneurial intentions, while students in Historical Studies, Philosophy, Procedural and Adjectival Law, and French have lower entrepreneurial intentions. Further interaction with university students revealed that students in the former departments have a higher entrepreneurial intention because most of these departments offer elective courses on entrepreneurship and provide a creative environment to develop ideas for new business start-ups. Maresch et al. [11] observe that tertiary educational programmes oriented towards entrepreneurship create higher levels of entrepreneurial intentions among university students in Chile. The findings are consistent with Maresch et al. [11] and Lopez and Alvarez [38]).

### 3.2 Section 2: Students’ decision on entrepreneurship based on level of education

This section presents the findings of statistical differences in students' entrepreneurial decisions based on their educational level. On entrepreneurial intention, it can be seen from Table 2 that master and undergraduate students have different entrepreneurial perspectives, as reflected in the item ‘I am ready to do anything to become an entrepreneur’ (Master = 468.43 and Undergraduate = 463.73, Mann Whitney U = 84228.50, and Z = −0.37, and p-value = 0.02); ‘My professional goal is to become an entrepreneur’ (Master = 470.99 and Undergraduate = 461.42, Mann Whitney U = 82878.00, and Z = −0.520, and p-value = 0.01); ‘I will make every effort to start and run my own business’ (Master = 470.99 and Undergraduate = 462.82, Mann Whitney U = 83610.00, and Z = −0.61, and p-value = 0.00); and ‘I am determined to create a business in the future’ (Master = 472.35 and Undergraduate = 462.28, Mann Whitney U = 83244.50 and Z = −0.76, and p-value = 0.00). The overall mean rank was recorded as 3732.91 with total mean score of 4.16. The mean of each category of respondents (undergraduate and postgraduate) was above 3.0 giving a p-value <0.05 in all the five categories of variables. This suggests that the majority of students, both post-graduates and undergraduates, have a strong desire to be entrepreneurs. The mean rank and mean of the master's degree students for various items is higher than that of undergraduate students. This implies that students with a higher educational level have a higher entrepreneurial intention, which is consistent with Saroia and Gao [39].

**Table 2:** Difference in students’ decision on entrepreneurship based on educational level (Master, n = 310; undergraduate, n = 620).

Components	Items	Level of Education	Response		
			Mean Rank	Mean, SD, Mann Whitney-U, Z-Value & *P*-value	
Entrepreneurial intention	I am ready to do anything to become an entrepreneur	Masters (n = 310)	468.43	Mean = 4.57 SD = 0.98	Mann Whitney U = 84228.50 Z = −.37 p-value = 0.02
		1. Undergraduate (n = 620)	463.73	Mean = 3.63 SD = 0.78	
	My professional goal is to become an entrepreneur	Masters (n = 310)	470.99	Mean = 4.65 SD = 0.99	Mann Whitney U = 82878.00 Z = −.52 p-value = 0.01
		2. Undergraduate (n = 620)	461.42	Mean = 3.74 SD = 0.81	
	I will make every effort to start and run my own business	Masters (n = 310)	470.89	Mean = 4.66 SD = 0.99	Mann Whitney U = 83610.00 Z = −.61 p-value = 0.00
		3. Undergraduate (n = 620)	462.82	Mean = 3.58 SD = 0.77	
	I am determined to create a business in the future	Masters (n = 310)	472.35	Mean = 4.89 SD = 0.99	Mann Whitney U = 83244.50 Z = −.76 p-value = 0.00
		4. Undergraduate (n = 620)	462.28	Mean = 3.56 SD = 0.77	
	**Total**		**3732.91**	**Mean=4.16**	
Attitude towards entrepreneurship	Being an entrepreneur implies mor e advantages than disadvantages to me	Masters (n = 310)	470.89	Mean = 4.66 SD = 0.99	Mann Whitney U = 82878.00 Z = −.52 p-value = 0.02
		5. Undergraduate (n = 620)	362.82	Mean = 3.45 SD = 0.95	
	If I had the opportunity and resources, I would like to start a firm	Masters (n = 310)	464.13	Mean = 3.48 SD = 0.75	Mann Whitney U = 84553.50 Z = −.05 p-value = 0.03
		6. Undergraduate (n = 620)	263.26	Mean = 2.87 SD = 0.91	
	Among various career options, I would rather be an entrepreneur	Masters (n = 310)	470.21	Mean = 4.53 SD = 0.96	Mann Whitney U = 83782.50 Z = −.436 p-value = 0.02
		7. Undergraduate (n = 620)	463.07	Mean = 3.19 SD = 0.69	
	**Total**		**2794.38**	**Mean=3.70**	
Subjective norms	My Parents will agree with me to start a new firm	Masters (n = 310)	468.81	Mean = 4.67 SD = 1.00	Mann Whitney U = 82507.00 Z = −.77 p-value = 0.21
		8. Undergraduate (n = 620)	254.71	Mean = 2.31 SD = 0.91	
	My Siblings will agree with me to start a new firm	Masters (n = 310)	364.13	Mean = 3.45 SD = 0.95	Mann Whitney U = 84553.50 Z = −.05 p-value = 0.17
		9. Undergraduate (n = 620)	263.26	Mean = 1.68 SD = 0.64	
	My friends will agree with me to start a new firm	Masters (n = 310)	362.38	Mean = 2.72 SD = 0.75	Mann Whitney U = 84431.00 Z = −.20 p-value = 0.24
		10. Undergraduate (n = 620)	265.97	Mean = 1.43 SD = 0.54	
	My teachers will agree with me to start a new firm	Masters (n = 310)	468.38	Mean = 4.49 SD = 0.96	Mann Whitney U = 82797.00 Z = −.73 p-value = 0.03
		11. Undergraduate (n = 620)	355.87	Mean = 3.01 SD = 0.85	
	**Total**		**2803.51**	**Mean=2.97**	
Perceived behavioural control	To start a firm and keep it working would be easy for me	Masters (n = 310)	486.37	Mean = 4.63 SD = 0.95	Mann Whitney U = 79726.00 Z = −1.61 p-value = 0.14
		12. Undergraduate (n = 620)	357.09	Mean = 2.61 SD = 0.73	
	I know the necessary practical details to start a firm	Masters (n = 310)	388.52	Mean = 2.98 SD = 0.77	Mann Whitney U = 79184.50 Z = −1.77 p-value = 0.21
		13. Undergraduate (n = 620)	256.29	Mean = 1.30 SD = 0.51	
	If I tried to start a firm, I would have a high probability of succeeding	Masters (n = 310)	379.83	Mean = 2.11 SD = 0.55	Mann Whitney U = 81365.50 Z = −1.34 p-value = 0.19
		14. Undergraduate (n = 620)	259.51	Mean = 1.41 SD = 0.54	
	**Total**		**2127.61**	**Mean=2.51**	
University Environment and Support	My department helps students to build the required network for starting a firm	Masters (n = 310)	356.60	Mean = 3.20 SD = 0.64	Mann Whitney U = 82980.50 Z = −.64 p-value = 0.13
		15. Undergraduate (n = 620)	268.11	Mean = 2.12 SD = 0.42	
	My department arranges for mentoring and advisory services for would-be entrepreneurs	Masters (n = 310)	384.08	Mean = 3.46 SD = 0.64	Mann Whitney U = 80300.50 Z = −1.52 p-value = 0.27
		16. Undergraduate (n = 620)	257.94	Mean = 2.31 SD = 0.51	
	My department provides a creative atmosphere to develop ideas for new business start-ups	Masters (n = 310)	365.11	Mean = 3.12 SD = 0.64	Mann Whitney U = 85012.00 Z = −.02 p-value = 0.18
		17. Undergraduate (n = 620)	264.69	Mean = 3.03 SD = 0.39	
	My department provides students with ideas to start a new business firm	Masters (n = 310)	368.84	Mean = 3.20 SD = 0.64	Mann Whitney U = 84125.00 Z = −.31 p-value = 0.16
		18. Undergraduate (n = 620)	263.58	Mean = 2.01 SD = 0.38	
	My department offers elective courses on entrepreneurship	Masters (n = 310)	479.35	Mean = 4.74 SD = 0.99	Mann Whitney U = 81487.00 Z = −1.14 p-value = 0.01
		19. Undergraduate (n = 620)	459.69	Mean = 4.10 SD = 0.77	
	My department arranges conferences and workshops on entrepreneurship	Masters (n = 310)	476.21	Mean = 4.35 SD = 0.91	Mann Whitney U = 82276.00 Z = −.88 p-value = 0.04
		20. Undergraduate (n = 620)	360.85	Mean = 3.11 SD = 0.90	
	**Total**		**4305.05**	**Mean=3.23**	

Master and undergraduate students also have different attitude towards entrepreneurship, as reflected by the item ‘Being an entrepreneur implies more advantages than disadvantages to me’ (Master = 470.89 and Undergraduate = 362.82, Mann Whitney U = 84553.50, Z = −0.05, and p-value = 0.02); ‘If I had the opportunity and resources, I would like to start a firm’ (Master = 464.13 and Undergraduate = 263.26, Mann Whitney U = 84553.50, Z = −0.05, and p-value = 0.03); and ‘Among various career options, I would rather be an entrepreneur’ (Master = 470.21 and Undergraduate = 463.07, Mann Whitney U = 83782.50, Z = −0.44 and p-value = 0.02). The overall mean rank (2794.38) is low among all the five variables. This suggests that students' attitude towards entrepreneurship is low. However, the total mean was high (3.70), implying that some students (Masters: 470.89), have exhibited more attitude towards entrepreneurship. A student who has a higher degree in the NUL have a more entrepreneurial attitude. This could probably be based on a general belief that master's degree programmes promote students' intellectual development, thinking abilities, and knowledge of the field of study compared to undergraduate programs [40].

In the case of subjective norms, the differences between master and undergraduate students are less apparent, as reflected by the item ‘my parents will agree with me to start a new firm’ (Master = 368.81 and Undergraduate = 254.71, Mann Whitney U = 82507.00, Z = −0.77, and p-value = 0.21); ‘my siblings will agree with me to start a new firm’ (Master = 364.13 and Undergraduate = 263.26, Mann Whitney U = 84553.50, Z = −0.05, and p-value = 0.17); ‘my friends will agree with me to start a new firm’ (Master = 462.38 and Undergraduate = 265.97, Mann Whitney U = 84431.00, Z = −0.20, and p-value = 0.24); and ‘my teachers will agree with me to start a new firm’ (Master = 468.38 and Undergraduate = 355.87, Mann Whitney U = 82797.00 and Z = −0.73, and p-value = 0.03). Subjective norms had overall mean ranks (2803.51) with total mean (2.97). The mean is below 3.0 and p-value >0.05 in most of the variables. Also, students studying master's degrees have high mean in most of the variables. This suggests that master's degree students in NUL had higher subjective norms than undergraduate students. Therefore, Williams et al. [19] concluded that the higher subjective norms people demonstrate, the higher the perceived control level of the behaviour, leading to high chances of performing a given behaviour.

Furthermore, the data on perceived behavioural control had a low overall mean of 2.51 for all variables in this category. This shows that there is no significant difference between Master's and Bachelor's students on the following items: ‘Starting a business and keeping it running would be easy for me’ (Master's = 486.37 and Bachelor's = 357.09, Mann Whitney U = 79726.00, 13 Z = −1.61, p-value = 0.14); ‘I know the practical details necessary to start a business' (Master = 388.52 and Undergraduate = 256.29, Mann Whitney U = 79184.50, Z = −1.77 and p-value = 0.21); and ‘If I tried to start a business, I would have a high probability of being successful’ (Master = 379.83 and Undergraduate = 259.51, Mann Whitney U = 81365.50, Z = −1.34 and p-value = 0.19.

Data about university environment and support showed high total mean (3.23) and high over all mean rank (4305.05) in all the variables presented in this category. This is evidenced in items such as; ‘my department arranges for mentoring and advisory services for would-be entrepreneurs” (Master = 384.08 and Undergraduate = 257.94, Mann Whitney U = 80300.50, Z = −1.523, and p-value = 0.13); ‘my department provides a creative atmosphere to develop ideas for new business start-ups’ (Master = 365.11 and Undergraduate = 264.69, Mann Whitney U = 85012.00, Z = −0.02, and p-value = 0.18); ‘my department offers elective courses on entrepreneurship’ (Master = 479.35 and Undergraduate = 459.69, Mann Whitney U = 81487.00, Z = −1.14, p-value = 0.01); and ‘my department arranges conferences and workshops on entrepreneurship’ (Master = 476.21 & Undergraduate = 360.85, Mann Whitney U = 82276.00, Z = −0.88, p-value = 0.04). The mean for master's degree students is above 3.0 indicating higher response than the undergraduate students. This further indicates that the university environment and support influence the decisions of graduate (master) students more than undergraduate students. This is because the university can create a platform that allows students to explore by performing a variety of tasks to develop their business potential and opportunities [40,41]. The high mean (above 3.0) could be attributed to other prevalent factors at the university like specialized programs, mentorship, networking opportunities, funding access, and practical experience. Nevertheless, the data show the extent to which the university is committed to availing itself to support students in the area of entrepreneurship.

It can therefore be concluded that postgraduate students (masters) have higher entrepreneurial decisions than undergraduate students. This is reflected in almost all the components of the TPB as presented in Table 3. Venturing into entrepreneurship can be difficult at the initial stages due to a lack of expertise. It takes a gradual process to master skills and develop more ideas. The higher we ascend the educational ladder, the more we meet new people, undertake courses (electives) on entrepreneurship, and become more exposed to new ideas and skills to start our businesses. Arguably, the NUL's master's degree programmes have the required resources that can help students to develop and experiment with entrepreneurial ideas with the help of experts and mentors than the undergraduate courses. This is consistent with the findings of Pulka, Rikwentishe and Ibrahim [42].

**Table 3:** Reliability and validity estimates.

Variable	Items	skew	Kurtosis	Standardized Loadings	AVE	α	CR
Attitude	ATT3	−0.906	−0.468	0.725			
	ATT2	−1.385	0.691	0.85			
	ATT4	−1.415	0.835	0.78			
	ATT5	−1.463	1.068	0.771	0.61274	0.862	0.863
Subjective Norms	SN5	0.883	−0.377	0.849			
	SN4	−0.516	−1.287	0.737			
	SN6	−0.214	−1.471	0.796	0.63253	0.836	0.837
Perceived Behaviour Control	PBC5	0.339	−1.098	0.868			
	PBC1	−0.026	−1.338	0.729			
	PBC6	0.124	−1.074	0.799	0.64109	0.838	0.842
Entrepreneurial Intention	ENT6	−1.475	1.577	0.903			
	ENT5	−1.549	1.111	0.879			
	ENT4	−1.837	2.374	0.742	0.71287	0.880	0.842

Note: AVE = Average Variance Extraction, α = Cronbach's alpha Reliability, CR= Composite Reliability.

### 3.3 Testing the relationship among the variables of the study

Structural equation modelling was used here to develop the relationship among the variables used for the study. The details of the structural equation model are shown and presented in Fig. 2.

### 3.4 Assessment of common methods bias

Guided by Fuller et al.‘s [43] recommendations and drawing on Harman's single-factor test, the study evaluated the possibility of common method bias. The results of Harman's single-factor test suggest that the total variance explained by a single factor is 20.79%. Since a variance explained by a single factor is far less than 50%, it is an indication of the absence of common method bias [44].

### 3.5 Reliability and validity of the measurement model

A study by Pesämaa et al. [45] maintains that reliability and validity are the hallmarks that guarantee the conceptual rigour of a measurement model. Thus, to ascertain the reliability of the constructs in the measurement model, the Cronbachs alpha method and composite reliability assessments were utilized as recommended by Robinson, Shaver and Wrightsman [46]. The results of the reliability analysis is as shown in Table 3. The Cronbach's alpha obtained in lieu of the reliability of items measuring attitide, subjective norms, perceived behavioural control and entrepreneurial intension was 0.862, 0.836, 0.838 and 0.880, respectively. Again, the composite reliability for attitide, subjective norms, perceived behavioural control and entrepreneurial intension was 0.863.0.837, 0.842 and 0.842, respectively. In line with Robinson, Shaver and Wrightsman [46], a threshold of 0.70 is sufficient to guarantee the reliability of a measure. It can therefore be descerned that all the measured constructs are reliable since they are higher than the 0.70 acceptable threshold.

The validity of the constructs in the measurement model were further estimated using convergent validity and discriminant validity as despited in Table 3, Table 4. For convergent validity, the obtained average variance extraction (AVE) for attitude, subjective norms, perceived behavioural control and entrepreneural intention which was 0.613, 0.633, 0.641 and 0.713, respectively, was above the acceptable threshold of 0.50 [47]. Hence, convergence validity can be inferred to exist for all the four variables [48]. Discriminant validity was also derived for the items by comparing the squared root of AVE for all the four variables with their latent variables correlations coefficients as directed by Saroia and Gao [49]. As seen in Table 4, a satisfactory discriminant validity is met since the squared root of AVE, which is across the tables diagonal, were comparatively higher than the latent variables correlation coefficients.

**Table 4:** Discriminant validity.

	Entrepreneurial Intention	Attitude	Subjective Norms	Perceived Behaviour Control
Entrepreneurial Intention	**0.844**			
Attitude	0.342	**0.783**		
Subjective Norms	−0.348	−0.202	**0.795**	
Perceived Behaviour Control	0.268	−0.016	0.12	**0.801**

### 3.6 Assessment of the structural model

The assessment of the structural model was an effort to ascertain the influence of attitude, subjective norms, perceived behavioural control on entrepreneurial intentions of students. To evaluate the structural model, the model fit indices were first estimated with AMOS software to indicate if the estimated model is good fit and the results is as shown in Table 5. According to Hair et al. [50], a structral model is said to be good fit when CMIN/df is lower than 0.5, the goodness-of-fit indices (GFI) is greater than 0.90, confirmatory fit index (CFI) is greater than 0.90, and Tuccker and Lewis index (TLI) is greater that 0.90. Hair et al. [50], posit that a structral model can be adjudged as fit if the root mean square error approximation (RMSEA) is between 0.05 and 0.08. In line with the preceding model fit indeces thresholds, the fit indeces for the our model as displayed in Table 5 were all within the accepted threshold: CMIN/df = 0.32, GFI = 0.95, CFI = 0.96, TLI = 0.945, RMSEA = 0.061.

**Table 5:** Model fit indices.

Model Fit Indices	Acceptable Threshold	Obtained Value	Comment
CMIN/df	<0.5	0.32	Good fit
GFI	>0.9	0.95	Good fit
CFI	>0.9	0.96	Good fit
TLI	>0.9	0.945	Good fit
RMSEA	>0.9	0,061	Good fit

The squared multiple correlation (R^2^) obtained for the structural model is 0.29 which indicates that 29% of the variance in students' entrepreneural intention is explained by their attitude, subjective norms, and perceived behavioural control. Results on the study's assessment of the relationship between attitude, subjective norms, perceived behavioural control, and students' entrepreneural intention are shown in Fig. 2 and Table 6.

**Table 6:** Summary of tested hypotheses.

Hypotheses	Standardized Estimates	T	p	Comment
Entrepreneurial Intention < --- Attitude	.285	8.544	***	Supported
Entrepreneurial Intention < --- Subjective Norms	−.377	−9.443	***	Supported
Entrepreneurial Intention < --- Perceived Behaviour Control	.236	8.999	***	Supported

Note: *** denotes significance at the one per cent level. Estimated using AMOS 21.

From Table 6, the influence of students attitude towards entrpreneurial intention was observed to be positive and statistically significant (β = 0.29, t = 8.544, p < 0.001). Hence, the first hypothesis is supported. Similarly, the impact of subjective norms on students entrpreneurial intention was detected to be negative and statistically significant (β = −0.33, t = −9.443, p < 0.001) and this indicates a support for the second hypothesis. Finally, the effect of perceived behavioural control on students entrpreneurial intention was noted to be positive and statistically significant (β = 0.31, t = 8.999, p < 0.001), which suggest that hypothesis three is supported.

According to Fig. 2 and Table 6, attitudes (0.285***) and perceived behavioural control (0.236***) were positive predictors of students' entrepreneurial intentions, while the subjective norm (−0.377***) was a negative predictor of students’ entrepreneurial intentions. The findings indicate that the more value students place on cultural norms surrounding entrepreneurship, the less they intend to start their own business after their university education, and vice versa. In addition, if students develop or gain more skills to manage their own businesses, their attitudes and intentions to start their own businesses increase.

## 4 Implications for practice

Based on the findings presented above, the following implications could be drawn to assist governments, educators and researchers who are interested in this field:

1. Given that NUL has a role to play in supporting the government's efforts to tackle youth unemployment by equipping students with the requisite entrepreneurship skillset, it is essential for NUL to streamline entrepreneurship courses as core components of undergraduate and graduate programmes. Incorporating entrepreneurship courses into the curriculum will inspire students who intend to go into entrepreneurship with business ideas that can serve as the basis for starting a business. Although students pursing programmes such as business management that are entrepreneurially inclined have high entrepreneurial intentions, streamlining entrepreneurship courses as core components of university programmes can spur entrepreneurial interest among students pursing non-business management programmes such as French, thereby boosting their entrepreneurial intentions and increasing their risk-taking abilities to engage in entrepreneurship. This can go a long way toward addressing Dungey and Ansell [12] concern that the lack of entrepreneurial and skill-developing components in Lesotho's education is partly the cause of low entrepreneurial intentions among its youth.
2. Having realised from the study that students who pursue university programmes that are entrepreneurial in nature, such as business management, develop positive entrepreneurial intentions, it is imperative for the government of Lesotho to provide the enabling and conducive environment for such students to put their intentions into reality. Government support in the form of financing start-ups and creating an innovation and entrepreneurship hub for commercializing and scaling up business ideas would contribute to the realization of the entrepreneurial intentions of university students and reduce unemployment after graduation.
3. The results from the study indicate that subjective norms have significant negative implications for students' entrepreneurial intentions. To mitigate the negative influence of subjective norms, it is imperative for educational institutions and curriculum developers to engage key stakeholders such as traditional and religious leaders, among others, who can help influence and change societal perceptions and norms. Such leaders command power and respect and can thereby play an instrumental role in changing social values and norms associated with entrepreneurship. Similarly, it is essential for the government to champion mass awareness among society, and most importantly, parents, on the value of entrepreneurship education for Lesotho's development.
4. It is recommended that professional development programmes be organized periodically for lecturers in the various departments to help them revitalize their knowledge and skills in entrepreneurship. As students in departments like Historical Studies, Philosophy, Procedural and Adjectival Law, and French were rated as having lower entrepreneurial intentions, lecturers in these departments must strengthen professional development programmes. We recommend that NUL lecturers should be resourced financially, intellectually, and in facility aspects to be able to guide and motivate students to participate in entrepreneurial ventures. This can be achieved if lecturers are empowered to organize workshops, symposiums, and forums for students to develop business ideas and talents. There is also the need to strengthen university-industry relations, which can serve as a springboard for the acquisition of practical and field-based experience and skills required for starting and managing a business among students.

## 5 Conclusion

It is evident from the findings that entrepreneurial intention is influenced by diverse factors, as supported by the TPB. Students' entrepreneurial intentions are differentiated by the programmes they pursue at university, with students in departments like Business and Management Development, Business Administration, Economics, Nutrition, and Pharmacy showed higher entrepreneurial intentions than their counterparts in Historical Studies, Philosophy, Procedural and Adjectival Law, and French. In addition, postgraduate students have higher entrepreneurial intentions than undergraduate students. Further, students’ entrepreneurial intentions are determined largely by perceived behavioural control, followed by subjective norms and attitudes towards entrepreneurship.

Findings from this study indicate promising entrepreneurial intentions among Lesotho youth. However, for students’ entrepreneurial intentions to materialise, it is important that the enabling environment and conditions are created for enhancing the implementation of youth entrepreneurial intentions. Hence, this study recommends the Lesotho government to go beyond the promotion of entrepreneurial education to providing financial and material support critical for starting and sustaining a business, particularly in the early stages of start-ups. A strong and reliable government-university-industry collaboration will be needed for the realization of robust youth entrepreneurship in Lesotho. For the NUL, is it essential that entrepreneurship courses are mainstreamed into university programmes to enable students, including those from non-business management backgrounds who have an interest in entrepreneurship, to develop the right skillset to turn ideas into business as well as start and manage a business.

## 6 Limitations and areas for future research

1. Given that the exploratory nature of this study and the bivariate research design are limitations of the study, future research may replicate this study using more robust statistical techniques and multivariate research designs that embrace other constructs such as prevailing economic conditions of countries, gender, and socio-demographic among others, that are not included in this study but may influence the ability of students to start and sustain a business. Also, qualitative studies can be conducted to enrich the findings of quantitative studies as the lived experiences of student entrepreneurs can put a spotlight on how government can address the challenges confronting star-ups in the country.
2. Recognizing the role of private universities in championing entrepreneurship among studies, future studies should not be limited to NUL but must include private universities and where possible conduct a comparative analysis.
3. Further studies should also explore how new curriculum and programmes can be developed, and existing one revised to enhance robust entrepreneurial intentions among university students in Lesotho.

## Production notes

### Author contribution statement

Christopher Dick-Sagoe: Conceived and designed the experiments; Performed the experiments; Analyzed and interpreted the data; Contributed reagents, materials, analysis tools or data; Wrote the paper.

Ka Yiu Lee, Ph.D.; Augustine Osei Boakye; Kofi Nkonkonya Mpuangnan: Peter Asare-Nuamah; Anna Dankwaah Dick-Sagoe: Analyzed and interpreted the data; Contributed reagents, materials, analysis tools or data; Wrote the paper.

### Data availability statement

Data will be made available on request.

## Declaration of competing interest

The authors declare that they have no known competing financial interests or personal relationships that could have appeared to influence the work reported in this paper.
